# Application of the identity disorder questionnaire AIDA+LoPF in adolescents with affective pathology and schizotypal disorder

**DOI:** 10.1192/j.eurpsy.2024.961

**Published:** 2024-08-27

**Authors:** M. Zvereva, N. Zvereva

**Affiliations:** clinical psychology, FSBSI MHRC, Moscow, Russian Federation

## Abstract

**Introduction:**

To assess the difference in the personality functioning of adolescents with schizotypal disorder and affective disorders, we used the AIDA + LoPF questionnaires, which are well established as questionnaires for identifying identity disorders in adolescents. We hypothesized that adolescents with affective pathology are much more likely to have identity disorders than adolescents with schizotypal personality disorder. Clinical assessment of diseases was carried out by psychiatrists using ICD-10

**Objectives:**

Adolescents with affective disorders -10 (F31), schizotypal disorder adolescents – 11 (F21). Age 12-18

**Methods:**

AIDA+LoPF questioners by authors K. Goth & K. Schmeck, Russian version by M. Zvereva & S. Voronova & N. Zvereva

**Results:**

The table presents statistical analysis data using the Mann-Whitney non-parametric test

**Table 1.** Significant scales of the AIDA questionnaire

SCALES AIDA	U-CRITERIA	P
TOTAL SCORE: IDENTITY INTEGRATION AIDA	86,000	0,02
Consolidating emotional self-experience	84,000	0,03
COHERENCE	86,500	0,02
CONSISTENCY IN SELF CONCEPTS	88,500	0,01
AUTONOMY, EGO-STRENGTH	88,000	0,02
INTEGRATING COGNITIVE SELF-EXPERIENCE	88,000	0,02

**Table 2.** Significant scales of the LoPF questionnaire

**SCALES LOPF**	**U-CRITERIA **	**P**
Identity	89,000	0,01
Coherence (Ego-strength)	88,000	0,02
SELF-DIRECTION	89,000	0,01
SELF CONGRUENCE	88,000	0,02
PURPOSEFULNESS	86,000	0,02

**Conclusions:**

We have obtained preliminary results that show a difference between the identity disturbance of adolescents with affective pathology and those with schizotypal disorder. Adolescents with affective pathology are much more likely to have various types of identity disorders than adolescents with schizotypal disorder. To clarify this, a larger sample and a wider range of disorders are required

**Disclosure of Interest:**

None Declared

